# Improved Disorder Prediction by Combination of Orthogonal Approaches

**DOI:** 10.1371/journal.pone.0004433

**Published:** 2009-02-11

**Authors:** Avner Schlessinger, Marco Punta, Guy Yachdav, Laszlo Kajan, Burkhard Rost

**Affiliations:** 1 CUBIC, Department of Biochemistry and Molecular Biophysics, Columbia University, New York, New York, United States of America; 2 Columbia University Center for Computational Biology and Bioinformatics (C2B2), New York, New York, United States of America; 3 Department of Biopharmaceutical Sciences, California Institute for Quantitative Biomedical Research, University of California San Francisco, San Francisco, California, United States of America; 4 NorthEast Structural Genomics Consortium (NESG), Department of Biochemistry and Molecular Biophysics, Columbia University, New York, New York, United States of America; 5 New York Consortium on Membrane Protein Structure (NYCOMPS), Department of Biochemistry and Molecular Biophysics, Columbia University, New York, New York, United States of America; Illinois Institute of Technology, United States of America

## Abstract

Disordered proteins are highly abundant in regulatory processes such as transcription and cell-signaling. Different methods have been developed to predict protein disorder often focusing on different *types* of disordered regions. Here, we present MD, a novel META-Disorder prediction method that molds various sources of information predominantly obtained from orthogonal prediction methods, to significantly improve in performance over its constituents. In sustained cross-validation, MD not only outperforms its origins, but it also compares favorably to other state-of-the-art prediction methods in a variety of tests that we applied. Availability: http://www.rostlab.org/services/md/

## Introduction

### Disordered regions come in different flavors

Many genes in higher organisms encode proteins or protein regions that do not adopt well-defined, stable three-dimensional (3D) structures under physiological conditions in isolation. These proteins are commonly labeled as *intrinsically disordered*, *unfolded,* or *natively unstructured proteins* [1], [2], [3]. Different words reflect differences in the underlying biophysical traits of these regions.

The assignment of *disordered* or *unstructured* regions is problematic, since by definition, these regions consist of an ensemble of rapidly inter-converting conformers that we cannot visualize. One way to circumvent this problem is by measuring biophysical characteristics that are associated with the lack of ordered 3D structure. Many techniques monitor properties such as distances between atoms, hydrodynamic features, and local or global changes in the environment of the atoms [4], [5], [6]. Since different experimental techniques capture different aspects or types of protein disorder, they occasionally do not agree on the assignments of these regions [7], [8]. For instance, a new experimental method is able to distinguish between molten-globule and other disordered states based on their susceptibility to 20S proteasomal degradation, providing operational definition for disorder. Results from this study suggested that unstructured regions in the cell are often protected from degradation by interaction with other molecules [8].

Disordered regions can be classified into three groups based on sequence features alone, where proteins from each group are identified by different experimental techniques [9]. Several new studies showed that disorder predictors trained on regions that were characterized as disordered by one experimental method are usually less accurate in predicting unstructured regions that were identified by a different technique [9], [10], [11]. Thus, there is no single gold standard for order/disorder assignment; instead, we need to use several experimental methods in concert [5], [12], [13], [14], [15], [16], [17].

We use the term “flavors” to refer to different types of disorder [9], [18] simply to indicate that we neither suggest a rigorous Aristotelian classification scheme, nor want to introduce any meaningful word for what appears a mesh of disorder. This mesh of *flavors* is accompanied by a variety of functional roles that increase organism complexity [11], [19], [20], [21], [22], [23], [24], [25], [26].

### Disordered regions have unique sequence characteristics

One of the main reasons for the predictability of unstructured regions is their amino-acid compositional bias. Unstructured regions are abundant in low complexity regions containing a reduced amino acid alphabet. They are usually depleted of hydrophobic and bulky amino acids, which are often referred to as “order promoting” residues [3], [27], [28]. Unstructured regions have a large solvent-accessible area, which explains why polar and charged residues, which favorably interact with water, are prevalent in these regions. Due to the high net charge of these regions, it was suggested that the unfolding is driven by charge-charge repulsion [3]. Other sequence-related biases in disordered regions include the high percentage of proline and frequent lack of regular secondary structure [9], [27], [29], [30]. The amino acid composition of disordered regions was also found to correlate with the length of disordered regions. For example, short disordered stretches are mainly negatively charged whereas long unstructured regions are either positively or negatively charged, but on average, nearly neutral [27].

Two types of short amino acid patterns are highly abundant in disordered regions: a proline-rich pattern and a (positively or negatively) charged pattern [31]. Interestingly, many of these proline-rich motifs in unstructured regions are important for protein-protein interactions. For instance, the polyproline-II (PPII) helix is a ubiquitous helical structure motif that is found in extended conformation and is abundant in molecular recognition features (MoRF) of unstructured regions [32]. The sequence-conserved unstructured motif P-X-X-P (where X is a variable amino acid) in the SH3 domain is important for mediating protein-protein interactions [33]. Numerous linear motifs mediate a variety of functions including protein localization, post-translational modifications and protein-protein interactions [34]. It has been estimated that ∼85% of the linear motifs from Eukaryotic Linear Motif (ELM) database are located within disordered regions [34], [35]. A recent study demonstrated the link between linear motifs and the putative mechanism for the interaction between unstructured regions and their partners [33].

### Prediction methods capture many different aspects of disorder

Some methods focus on the fact that unstructured regions tend to have low hydrophobicity/high net-charge [3], [36], high loop content [37], and few stable intra-chain contacts [38], [39]. One major limitation of methods using this approach is that they are protein- and position- independent. That is, they only depend on the amino acid composition of the sequence and do not take into account the specific order of the residues. This simplification ignores the important roles that some disordered regions play in target recognition by forming highly specific electrostatic interactions and hydrogen bonds upon folding and binding to substrates [40], [41], and through the use of conserved motifs [33], [34].

Several advanced methods attempt to capture complex relationships between sequence and disorder by using machine-learning algorithms optimized to discriminate between well-structured and unstructured regions [18], [42], [43], [44], [45], [46], [47]; these methods are usually very good for what they are trained for, for example, the identification of residues that do not appear in electron density maps of X-ray structures [46], [48], [49], [50]. Many of these methods use protein-specific sequence properties such as profiles of evolutionary exchanges. One limitation of methods based on machine learning is that they are prone to over-optimization when developed on data sets as small as the Database of Protein Disorder (DisProt) or as specialized as missing coordinates from the Protein Data Bank (PDB). Performance assessments should therefore be taken with a grain of salt.

Due to the fuzzy definitions of mobility/disorder/flexibility, some predictors focusing on different aspects of protein mobility can sometimes capture protein disorder [11], [37], [51], [52], [53], [54]. For instance, the method Wiggle was optimized to identify functionally flexible regions and captures some aspects of disorder [53]. Our group identified long regions with no regular secondary structure (NORS), i.e. ≥70 sequence-consecutive surface residues depleted of helices and strands [55]. NORS regions share many cellular, biochemical and biophysical properties with long unstructured regions in proteins [54], [55]. Loops with high B-factors also correlate with disorder [37], [49]. In fact, a recent study demonstrated that PROFbval, which was trained on regions with high normalized B-factors from the PDB, accurately predicted the long unstructured region in the adaptor protein GAD [56]. Another method, NORSnet, distinguishes between long (>30 residues) loops that are well-structured and those that are disordered [11]. While most of these methods are not optimal for the identification of the “average” disorder, they are usually optimized on data sets that are very large and are not biased by current experimental means of capturing disorder. Thus, they reach into regions in sequence space that are not covered by the specialized disorder predictors [11], [57], [58].

Some methods combine more than one approach where the combined methods typically outperform individual approaches. For instance, one method employs a neural network trained on residues missing from electron density maps and on residues in high B-factor loops [42]. A recently developed method is based on the consensus of the distributions of charge-hydropathy values and disorder prediction scores to predict proteins that are mostly disordered [10]. Another predictor uses two different prediction methods, each optimized on unstructured regions of different lengths [28]. Recently, we developed a method that combines inter-residue internal contacts with pairwise energy potentials and accurately predicts long and functional unstructured regions [59].

### Better methods still urgently needed

The unraveling of the phenomenon of disorder continues. We need more and better specialists, i.e. methods that identify specific types of disorder and through this facilitate the functional and structural interpretation such predictions. We also need more accurate generalists, i.e. methods that perform best for most types of disorder. Finally, despite the variety of current prediction methods, some aspects of disorder remain untapped, demonstrated by the observation that if a new experimental technique for identifying disorder comes along, existing methods fail impressively (GT Montelione, unpublished). Some methods account for these demands by combining original methods [28], [42], [59]. As for other prediction tasks, it has been demonstrated that a simple combination of just few orthogonal methods improved accuracy over all its original sources [10].

In this work we hypothesized that a combination of several orthogonal methods will capture many types of disorder at improved performance without sacrificing the distinction of the type of disorder that is detected. We first showed that even a simple arithmetic average over different methods slightly improved over the best method confirming and expanding previous observations [10], [11], [28], [42], [59]. We topped this significantly by combining the output from various prediction methods with sequence profiles and other useful features such as predicted solvent accessibility, secondary structure and low complexity regions. The new method, MD (Meta-Disorder predictor), significantly outperformed each of its constituents on average and in our tests also topped commonly used top-of-the-line methods such as RONN, IUPred and the VSL2 series of prediction methods.

## Results and Discussion

### Simple averaging over output improved over best individual method

**Figure 1:**
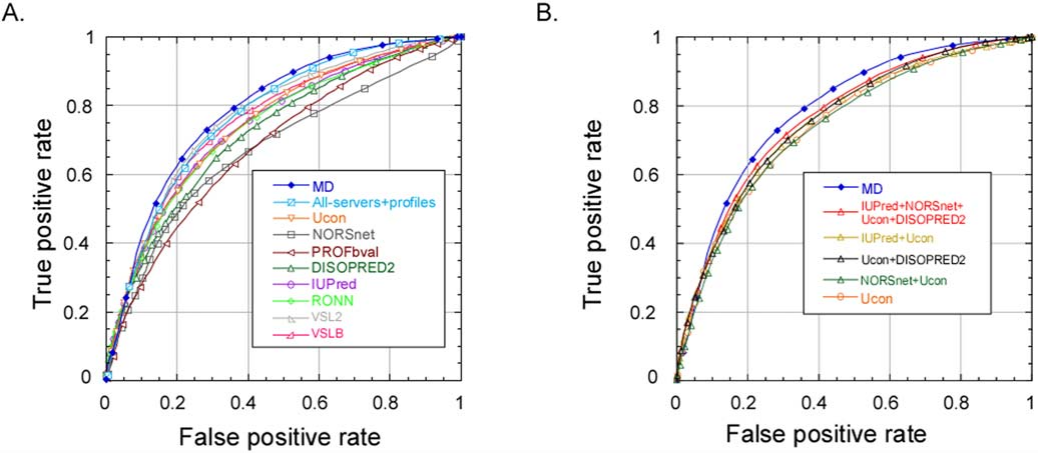
Per-residue performance on sequence-unique DisProt subset. (A) The final method MD (blue filled diamonds), which uses neural networks to combine the output of other methods with sequence profiles and other sequence features, is significantly more accurate than the methods that it uses as input such as NORSnet (dark gray) and DISOPRED2 (dark green) as well as other popular predictors such as IUPred (purple), RONN (light green), VSL2B (pink) and VSL2 (light gray). Other VSL2 models resulted in AUCs ranging the values obtained by VSL2B (sequence based) and VSL2 (sequence+secondary structure+profiles). Note that the VSL methods were trained on DisProt. Since we tested that method on essentially the same data set without cross-validation, our results are likely to over-estimate the performance of the VSL methods. Using additional sequence features also improved over using only the output from other methods and profiles (light blue open diamonds). (B) We compared methods that would result from simply averaging over the output of original prediction methods (triangles). Most averages were better than the best original method (here Ucon, orange circle). Our final neural network-based method, MD, significantly outperformed others throughout almost the entire ROC-curve.

First, we calculated the arithmetic average over the raw output of four disorder prediction methods: DISOPRED2 (Support Vector Machine based prediction of missing coordinates in X-ray structures), IUPred (prediction of unstructured regions based on pairwise statistical potential), NORSnet (prediction of unstructured loops) and Ucon (specific contact based prediction method). The resulting method was better than any of the original methods (AUC>0.77, Fig. 1A). Even an average compiled exclusively over the most accurate individual method (Ucon) and a less accurate but quite orthogonal method (DISOPRED2) improved slightly (AUC>0.76, Fig. 1B). The main reason for the improvement was the difference in their predictions [59]. A combination of accurate but similar methods (Ucon and IUPred) hardly improved on its components (AUC = 0.76). Not all simple combinations yielded better predictions, e.g. the average over Ucon and NORSnet (AUC = 0.75) did worse than Ucon. These results were particularly important in light of researchers who are confused by the plethora of existing prediction methods and respond by compiling averages, which is not always a good idea.

### Final method MD better than simple averaging

We then input to neural networks the results from the above four servers along with the output of a method predicting flexibility (PROFbval), and sequence profiles. This method outperformed any of its constituents (AUC = 0.78, Fig. 1A) as well as the best simple average over the original four methods (AUC = 0.77, Fig. 1B). Then, we trained our final method which also included explicit predictions of secondary structure, solvent accessibility and other sequence properties (Methods). This final meta-disorder prediction method topped the previous ones considerably (AUC = 0.80, Fig. 1B). The method, MD, significantly outperformed its components (NORSnet, PROFbval, Ucon and DISOPRED2) as well as other predictors, such as IUPred and RONN [60], which have been demonstrated to be rather accurate [39], [49]. MD also outperformed all VSL2 methods that we tested, including VSL2 (AUC = 0.77), one of the most accurate predictors at the 7^th^ Critical Assessment of methods of protein Structure Prediction (CASP7) [49], [61]. VSL2 itself is a meta-predictor that combines different approaches [28], [62]. Overall, our results show that averaging over many tools can go wrong, and there is always a prediction available that is considerably better than the best average (Fig. 1B). Note that similar results were observed for a subset of proteins that did not share homology using a stricter cutoff (HSSP-value<0, Fig. S1).

### Final method best for all flavors of disorder captured by other methods

**Figure 2:**
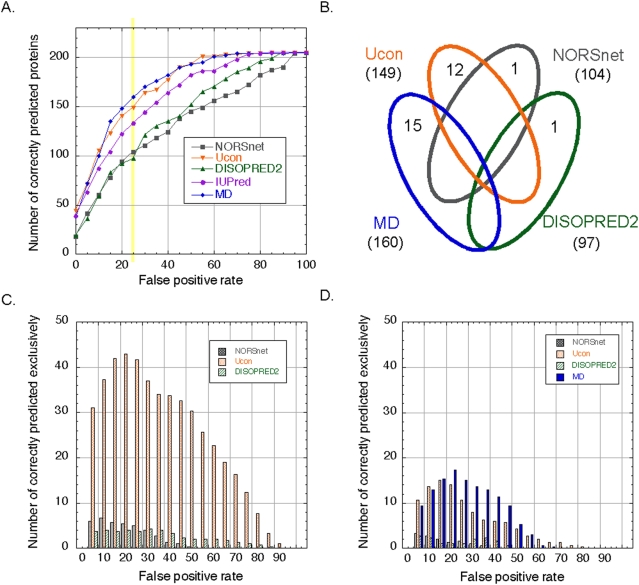
Per-protein performance on long disordered regions. Data set: 205 DisProt proteins with at least one long (>30 residues) disordered region. (A) Our final method MD identified more true positives than the other methods at most of the false positive rates. (B) The results for false positive rates ≤0.25 (yellow bar) are presented in the Venn diagram. The numbers in parentheses correspond to the y-axis values of the points in the yellow column in graph (A). (C+D) This is the same data as for (A) except that we only considered the subset of proteins correctly predicted exclusively by the method shown, i.e., proteins with long disordered regions that no other method captured. Due to low counts, we smoothed values by running averages over three percentage points. In (C) the panels represent the proteins that are unique if MD is not included in the overlap calculation, whereas in (D) the panels represent the proteins that are unique when MD is included. The number of unique predictions is substantially smaller when including MD suggesting that MD not only yielded a good average but also captured all types of disorder.

MD was best in terms of per-residue performance, but it also distinguished best between proteins with and without long (>30 residues) disordered regions: at a prediction threshold with an estimated false-positive rate <0.25, MD correctly identified 160 proteins, while NORSnet, Ucon, DISOPRED2 and IUPred identified 104, 149, 97 and 133 proteins, respectively (yellow column in Fig. 2A and Venn diagram in Fig. 2B). We confirmed this trend for a dataset that was compiled using more stringent cutoff for homology (HSSP-value<0, Fig. S2). IUPred and Ucon were previously established to be very accurate in the distinction between disordered and well-ordered long regions. As MD was trained to capture the entire length spectrum, i.e. also short regions with disorder, it was particularly encouraging that MD competed successfully with those two original methods. The question remains whether MD is just zooming into the type of disorder that is most commonly captured by today's tools.

Not all prediction methods capture the same flavor of disorder [11], [59]. Here, we analyzed the set of proteins correctly identified at false positives rates ≤0.25 to have at least one long disordered region (Fig. 2A, yellow column). Most of the proteins (145 of 160 proteins) identified by MD were also predicted by at least one of the other methods. Surprisingly, MD identified 15 proteins that all other methods missed (*unique predictions*, Fig. 2B). In contrast, NORSnet and DISOPRED2 had relatively low number of unique predictions; this is partially due to the fact that these two methods overlap with each other: NORSnet predicts unstructured loops and DISOPRED2 predicts residues missing from the electron density map in X-ray structures, which are often flexible loops.

One limitation of Venn diagrams is that they may hide trends because they represent predictions for a single cutoff. We addressed this problem by plotting the per-residue false positive rate against the number of unique proteins, i.e. proteins that were not identified by any of the other methods (Fig. 2C–D). We first compiled unique predictions for only three methods (Ucon, NORSnet and DISOPRED2) and then compared this to the unique predictions upon including MD. Including MD shrunk the number of unique predictions considerably, supposedly because it captured some features of each of the three original methods (Fig. 2C–D). While excluding predictions by any method is likely to drop the total number of correctly predicted proteins, we found that when excluding proteins identified by MD this number had shrunk the most (Fig. S3). This view again revealed that MD captured surprisingly many disordered regions that none of the other methods had identified. The downside of this result was that for those cases, we no longer have evidence as to which flavor of disorder is predicted; this makes interpretations about the structural and functional impacts of the region more challenging. On the other hand, MD shares this occasional disadvantage with many prediction methods [10]. Moreover, one simple aspect of disordered regions is their length. Overall, the length distribution predicted by MD was very similar to the one in observed regions (Fig. S4). Limitation of some of the experimental methods characterizing disorder and computational methods serving as input features for MD may have led to apparent over-prediction of short stretches and under-prediction of long regions (Fig. S4).

### Stronger predictions of disorder more accurate

**Figure 3:**
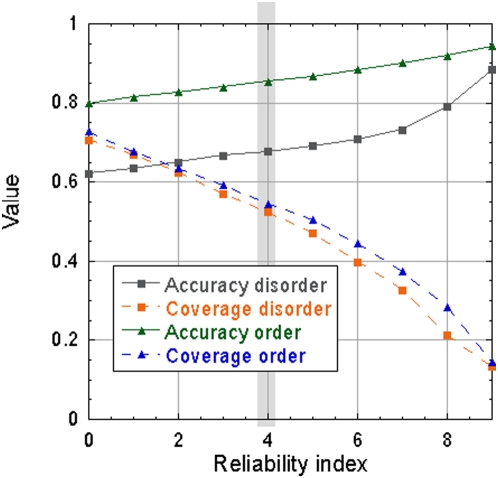
Reliability index allows focusing on more accurate predictions. The normalized output of MD was converted into a reliability index that reflects the prediction strength. Different performance measures (Eqn. 1 and 2) were calculated and averaged over the six sets using the default cutoff defining positive prediction. Stronger predictions (higher reliability indices) were, on average, more accurate, e.g. if a user looked only at residues predicted at RI≥4, then she or he would expect to find about 52% of all disordered residues at that level, and over 68% of the residues identified at that level would be correct (marked by gray column). Note that one limitation of using DisProt is that the per-residue assignment of long unstructured regions can be inaccurate as some experimental techniques characterizing disorder may only capture global properties of the protein resulting mislabeling of the whole domain or protein as disordered.

The distribution of the normalized method output (compiled as the difference between the two output units) indicates that disordered residues tend to have higher output values than ordered residues (Fig. S5, Supporting Online Material). Therefore, we converted this normalized output into a reliability index (RI), and found that this measure correlated well with accuracy and coverage (Fig. 3). In this analysis we focused on residues from long unstructured regions (>30). For example, ∼52% of the disordered residues from long unstructured regions in the DisProt data set were predicted at RI≥4 (*coverage* in Eqn. 1); at that level, the prediction accuracy was>68%, compared to 62% for all residues. The method is particularly accurate for ordered residues. For instance, for the same reliability index, ∼55% of the residues that are not located in long unstructured regions were predicted at ∼85% accuracy (*coverage ordered* and *accuracy ordered* in Eqn. 2).

### MD output provides hints for the predicted disordered region type

Although it is evident from Fig. 2 that MD predicts new unstructured regions, it is not clear what regions MD captures that other methods “miss”. Ultimately, the achievement of MD over its constituents appears to be one of slightly moving thresholds. In the context of analyzing entire proteomes as well as structural and functional genomics, methods that move cases from “may be disordered” to “clearly disordered” may matter very much. Note that the ROC curves (Fig. 1, Fig. 2A) indicate relatively sharp transitions, i.e. moving the threshold slightly may identify hundreds of proteins in human alone that might fall out of the analysis without MD.

The question remains as to what types of disorder MD pulls out. Are they “salvaged ones” loopy-like (as identified by NORSnet)? Or are they low in contact propensity (as predicted by Ucon)? If we had used a simple neural network that only uses the output from other methods as input, we could easily analyze the contribution of the input to the final decision. However, we found that such a simple network did not improve importantly enough over simple averaging, and therefore included a lot of other information. We are not aware of any analysis that succeeded in gaining understanding from the “rules” contained in levels of such complexity in real-life applications of networks. Put simply: when problems are so complex that their solutions need very high levels of complexity, it is more difficult to fool ourselves into believing that we understand the dominant sources.

**Figure 4:**
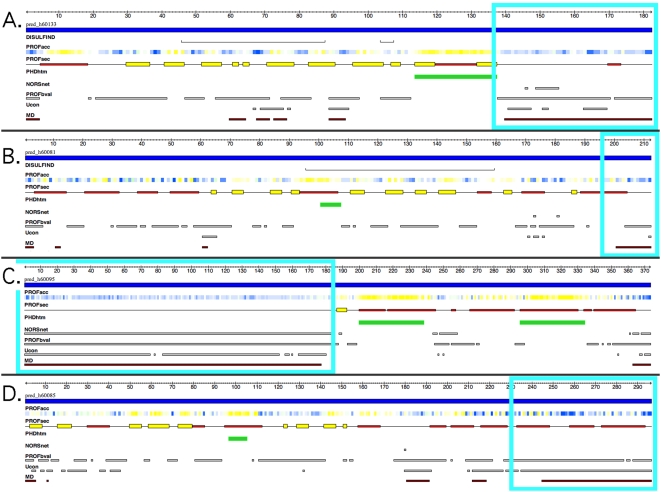
MD predictions demonstrated by specific examples. Predicting disorder and other sequence features using the MD server through the PredictProtein web-interface for protein sequence analysis (Methods) [75], [76]. (A) NORSnet and Ucon predict some signal for the presence of disordered region in the C-terminal domain of T-cell surface glycoprotein CD3 gamma chain (DP00508) [77], while MD correctly predicts the whole domain to be disordered. (B) Similar results were obtained for the C-terminal domain of *E. Coli* Alkylmercury Lyase (DP00575) [78]. (C) The signaling molecule Nogo-B (DP00524) [79] contains disordered N-terminal, which was captured by MD. PROFsec and NORSnet predictions suggest that this region is long disordered loop. (D) The C-terminal domain of the ribosomal protein L5 (DP00579) [80] is disordered. While PROFsec predicted this region to be helical (red rectangles), Ucon identified it as disordered, probably due to small number of internal contacts. MD agreed with Ucon output and correctly predicted this region to be disordered.

An *ad hoc* approach is to simply provide the raw output of all constituent prediction methods, some of which allow very clear interpretations of the flavor of disorder that they pick up. In the examples shown in Fig. 4, we analyzed predictions by MD, as well as some of its constituents and other sequence features including secondary structure and solvent accessibility. None of these recently annotated disordered regions has been used to train MD or any of its constituents. For both the C-terminal domains of cell-surface glycoprotein CD3 gamma chain and alkylmercury lyase, Ucon and NORSnet gave some signal of disorder (Fig. 4A–B), thereby correctly predicting some parts of the disordered regions. In both cases MD captured the whole disordered region. This observation is not surprising; while MD does not define a completely new type of disordered region, it averages scores from several prediction methods and other sequence properties to define a new, refined score predicting disorder. Although one can argue that by changing the thresholds of the other methods they can also predict MD-identified regions, we hypothesize that MD can do it effectively in an automatic manner. Finally, we demonstrate how by combining results from secondary structure prediction, different disorder predictors and MD, one can estimate the type of the predicted disorder region (Fig 4C–D). For instance, as illustrated in Figure 4D, NORSnet, predicts the protein to be entirely lacking unstructured loops and PROFsec, a profile neural network based method predicting secondary structure, predicts the disordered region to be mostly helical. Ucon, which focuses on identifying disordered regions with low contact-density, predicts the protein to have a disordered region. In this case, MD correctly predicted the Ucon-like disordered region.

### Conclusions

We demonstrated that methods predicting disorder based on different concepts identified very different “flavors” of disorder. Two extreme examples were contributed by the results of methods such as NORSnet and DISOPRED2 on the one side and IUPred and Ucon on the other side. While the field will need more specialized methods that capture regions in the space of disordered sequences that remain untapped, here our goal was the development of the best generic prediction method. In all our comprehensive tests, we amassed data supporting the notion that we succeeded in implicitly extracting the best of each specialist and in carving this into an excellent generalist, dubbed MD. MD not only performed best in terms of per-residue and per-protein accuracy/coverage, but it also identified unique regions that had been missed by ALL the original methods that we analyzed, i.e. it somehow intruded into the untapped region of sequence space. Nevertheless, the downside of averaging is always that some pearls discovered by the original methods can be lost when only considering the average, i.e. MD. Therefore, it is probably best to use the most reliable predictions from many methods on top of MD.

## Materials and Methods

### DisProt data set

We used all residues that were shown by at least one experimental technique to be in disordered regions according to DisProt version 3.4 [7] as positives, and all other residues in those proteins as the negatives. Unlike in our other studies, we used residues from disordered regions of all lengths (expecting the meta-predictor to pick up all types of disorder). Note that DisProt regions are on average longer than regions of missing residues from X-ray structures, and have different amino acid composition (data not shown).

From the initial set of 460 proteins we discarded 60 proteins with >780 residues as these could not be handled by all of the methods we tested. From the remaining set, 17 more proteins crashed when applying at least one of the predictors in this study, and were also discarded. We generated sequence-unique subsets through UniqueProt [63] ascertaining that the pairwise sequence similarity between any pair of proteins corresponded to HSSP-values<10 [64], [65] which translated to <31% pairwise sequence identity for >250 aligned residues. Alignments were generated by three iterations of PSI-BLAST [66] searches against UniProt using our standard protocol for the generation of profiles [67]. The entire data set included 298 sequence-unique proteins with 27,117 disordered (positives) and 61,118 well-structured (negatives) residues. Our results were qualitatively similar for sequence-unique filtering at HSSP-values<0 (i.e., 21% pairwise sequence identity for >250 aligned residues); however, for that number only 135 proteins remained in the DisProt data set.

### Neural networks: training, cross-training and testing

We randomly divided the sequence-unique data set into six equally sized groups, using proteins from four groups for training (optimization of junctions in the neural networks), one for cross-training (optimization of general network parameters, including “stop-training”), and one for testing (estimate performance). We then rotated through these sets so that each protein was used exactly once for testing, and averaged the performance measures over the six groups. All the results that we reported were valid for the independent testing sets.

### Input from prediction methods

In selecting the methods used as input to the Meta-disorder predictor (MD) we applied the following rationale:

1. Include the most unique methods: to prevent over-optimization for one particular type of disorder, we focused on methods that were based on different concepts.
2. Preference for in-house methods: this focus originated solely from considerations that had to do with the prospect of having to manage the resulting method for a considerable amount of time in environments of constant changes.
3. Preference for easily reproducible algorithms: methods that are based on simple concepts, such as the statistical potential based method IUPred [39] and the hydrophobicity/net-charge based method FoldIndex [3], [36] can easily be reproduced by anyone. Our resulting local versions of these methods were slightly less accurate than the originals when tested on our data sets.
4. Preference for methods that can be installed locally and can be used freely. Since one important aspect of protein disorder is the prediction of residues that are invisible in X-ray structures, we needed to use one of the methods that predict this aspect as input for our meta-predictor. Many machine learning based methods were optimized for residues missing from PDB structures [28], [42], [43], [44], [45], [46], [68]. Despite many differences, these methods overlap. Therefore, we decided to represent this class by the incorporation of one single method, namely DISOPRED2 [46]. We used DISOPRED2 for several reasons: it was one of the best methods according to the CASP6 disorder assessment [49], it installed easily locally, and DISOPRED2 is quite orthogonal to our in-house methods [11], [59].

### Neural network architecture

We trained standard feed-forward neural network with back-propagation and a momentum term [69]. Due to a significant difference in the number of positive and negative samples we used balanced training [69]. The input features for the network included properties that were shown to be correlated with protein disorder: (1) local properties such as predicted secondary structure, local sequence profiles, solvent accessibility, the presence of low complexity regions, and amino acid composition of a given sequence window length; (2) global properties such as the length of the sequence; (3) predictions from other servers that included the probability for a given residue to be disordered. These included NORSnet [11], DISOPRED2 [46], PROFbval [58], [70] and Ucon (where several models were implemented) [59]; (4) for the reproduction of predictors similar to the amino acid propensity based methods FoldIndex [3], [36] and IUPred [39], we calculated hydrophobicity/net-charge as described by Uversky [3] and estimated the energy of a local sequence window using a statistical potential, respectively. Note that we also trained a method that used as input only predictions from NORSnet, DISOPRED2, PROFbval, Ucon and sequence profiles without using any other sequence properties.

### Per-residue vs. per-protein performance

Many of the methods used as input to MD used DisProt and similar sets for parameter optimization. Monitoring per-protein prediction is more prone to over-optimization than monitoring per-residue performance as the set contains significantly fewer samples; it also may bias the results for predicting proteins with very short unstructured regions. In order to minimize this risk, we focused on per-residue predictions and only ultimately, assessed per-protein performance. We also validated the performance of MD on a subset of our set that was obtained using a more stringent criterion for sequence uniqueness, i.e., for HSSP-values<0. For the per-protein analysis, we used a sequence-unique subset of DisProt that consisted of 205 proteins with at least one long (>30 residues) disordered region, and again, validated the results on a set that was created using the more stringent criterion for sequence uniqueness.

### Assessing performance

We assessed performance on the DisProt data set. All results in the study were based on the sequence-unique subset; some data for the full set is provided in Supporting Online Materials. Receiver operating characteristic (ROC) curves were constructed by calculating FP (false positives) and TP (true positives) rates at different thresholds defining a positive prediction. The curves were then integrated in order to calculate the area under the curve (AUC). TP are unstructured residues experimentally observed AND correctly predicted; FP are structured residues that are predicted to be unstructured; TN (true negatives) are residues observed and predicted as well-structured, and FN (false negatives) are residues observed to be unstructured and predicted to be structured.

We also measured accuracy/specificity (Acc), coverage/sensitivity (Cov) and false positive (FP) rate by the standard formulas:
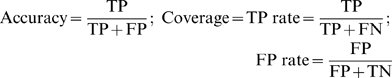
(1)In analogy, we computed the accuracy and coverage for the negatives, i.e., residues that there is no evidence for them to be disordered, thus we assume they are structured:
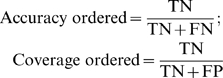
(2)


### Web-server

MD server provides results in text and graphical formats. To gain further insight into the nature of the predicted disordered region, the server also provides visual output of methods predicting different aspects of protein structure and function (Fig. 4). DISULFIND [71] is a method that predicts cysteine pairs found in disulfide bridges. Predicted pairs are marked by squared brackets connecting the positions of two residues along the protein sequence. PROFacc [72] is a method that predicts residue solvent accessibility. Predictions range from highly accessible (blue) to fully buried (yellow). PROFsec [69], [73] is a method that predicts secondary structure. Yellow rectangles represent predicted strands; red smaller rectangles represent alpha helices. PROFhtm [74] is a method that predicts transmembrane helices (green rectangles). The remaining methods predict different aspects of disorder as described in the text.

## Supporting Information

Figure S1 Per-residue performance on sequence-unique DisProt subset using a stringent homology cutoff. ROC curves were compiled using a set with a stricter cutoff for homology redundancy - HSSP-values are <0. The final method MD (blue filled diamonds) that uses neural networks to combine the output of other methods with sequence profiles and other sequence features, is significantly more accurate than the methods that it uses as input such as NORSnet (gray) and DISOPRED2 (dark green) as well as other popular predictors such as IUPred (purple) and RONN (light green).
(0.56 MB TIF)

Figure S2 Per-protein performance on long disordered regions. Data set: 86 DisProt proteins with at least one long (>30 residues) disordered region. This set was compiled using more stringent cutoff for homology (HSSP-values<0). Our final method MD identified more true positives than the other methods at most of the false positive rates. Note that this set is much smaller than the one compiled using HSSP-values<10 that the error margins are significantly higher.
(0.56 MB TIF)

Figure S3 Per-protein performance on long disordered regions when excluding proteins identified by the different methods. Data set: 205 DisProt proteins with at least one long (>30 residues) disordered region. Each line represents the performance when taking protein regions that were correctly identified as disordered by at least one of the methods, while excluding proteins identified by one method. For example, the worst performing combination of three methods is when we did not include MD predictions (blue filled diamonds).
(0.59 MB TIF)

Figure S4 Distribution of observed vs. predicted disordered regions lengths. The fractions of residues that originated from disordered regions from different lengths are plotted. More than 50% of the observed disordered residues originated from very long unstructured regions - regions that are longer than 220 consecutive unstructured residues (dark blue squares), and only about 35% of the predicted residues originated from very long unstructured regions (light blue triangles). Overall, the predictions and observations differed significantly for the two extreme ends of the distribution: MD significantly over-predicted short regions (<30 residues) and significantly under-predicted very long regions. This large difference could be attributed to two main factors; first, among MD's most useful input features was the disorder probability predicted by DISOPRED2. While DISOPRED2 was trained on X-ray disorder, it identifies many short regions as disordered that some were predicted as such by MD as well. This observation gives further evidence that MD captured the flavor of disorder predicted by DISOPRED2. Future improvements of MD may include filtering out very short and isolated predicted stretches. Second, some of the experimental methods characterizing unstructured regions are not accurate enough to determine disorder in a resolution of a few residues. In fact, experimental techniques such as circular dichroism (CD) and analytical ultracentrifugation can only assign disorder at the protein or domain level.
(0.42 MB TIF)

Figure S5 Distribution of method values. The difference between the two neural network output units (one coding for disorder, the other for ordered) was normalized to values ranging from 0 (ordered) to 100 (disordered). Some disordered residues have very low values, i.e. are predicted strongly as well-ordered. These might just be bad, generic prediction mistakes or problems in the original data. Interestingly, residues from E. coli tend to be very low and residues from H. sapiens follow similar distribution to our set.
(0.63 MB TIF)

## References

[1] Dyson HJ, Wright PE (2005). Intrinsically unstructured proteins and their functions.. Nat Rev Mol Cell Biol.

[2] Dunker AK, Obradovic Z (2001). The protein trinity-linking function and disorder.. Nature Biotechnology.

[3] Uversky VN, Gillespie JR, Fink AL (2000). Why are “natively unfolded” proteins unstructured under physiologic conditions?. Proteins: Structure, Function, and Genetics.

[4] Eliezer D (2007). Characterizing residual structure in disordered protein States using nuclear magnetic resonance.. Methods Mol Biol.

[5] Bracken C, Iakoucheva LM, Romero PR, Dunker AK (2004). Combining prediction, computation and experiment for the characterization of protein disorder.. Curr Opin Struct Biol.

[6] Tompa P (2002). Intrinsically unstructured proteins.. Trends Biochem Sci.

[7] Vucetic S, Obradovic Z, Vacic V, Radivojac P, Peng K (2005). DisProt: a database of protein disorder.. Bioinformatics.

[8] Tsvetkov P, Asher G, Paz A, Reuven N, Sussman JL (2007). Operational definition of intrinsically unstructured protein sequences based on susceptibility to the 20S proteasome.. Proteins.

[9] Vucetic S, Brown CJ, Dunker AK, Obradovic Z (2003). Flavors of protein disorder.. Proteins.

[10] Oldfield CJ, Cheng Y, Cortese MS, Brown CJ, Uversky VN (2005). Comparing and combining predictors of mostly disordered proteins.. Biochemistry.

[11] Schlessinger A, Liu J, Rost B (2007). Natively Unstructured Loops Differ from Other Loops.. PLoS Comput Biol.

[12] Mittag T, Forman-Kay JD (2007). Atomic-level characterization of disordered protein ensembles.. Curr Opin Struct Biol.

[13] Uversky VN (2002). What does it mean to be natively unfolded?. Eur J Biochem.

[14] Uversky VN (2002). Natively unfolded proteins: a point where biology waits for physics.. Protein Sci.

[15] Receveur-Brechot V, Bourhis JM, Uversky VN, Canard B, Longhi S (2006). Assessing protein disorder and induced folding.. Proteins.

[16] Snyder DA, Chen Y, Denissova NG, Acton T, Aramini JM (2005). Comparisons of NMR spectral quality and success in crystallization demonstrate that NMR and X-ray crystallography are complementary methods for small protein structure determination.. J Am Chem Soc.

[17] Yee AA, Savchenko A, Ignachenko A, Lukin J, Xu X (2005). NMR and X-ray crystallography, complementary tools in structural proteomics of small proteins.. J Am Chem Soc.

[18] Obradovic Z, Peng K, Vucetic S, Radivojac P, Brown CJ (2003). Predicting intrinsic disorder from amino acid sequence.. Proteins: Structure, Function, and Genetics.

[19] Tompa P, Szasz C, Buday L (2005). Structural disorder throws new light on moonlighting.. Trends Biochem Sci.

[20] Dunker AK, Cortese MS, Romero P, Iakoucheva LM, Uversky VN (2005). Flexible nets. The roles of intrinsic disorder in protein interaction networks.. Febs J.

[21] Dosztanyi Z, Chen J, Dunker AK, Simon I, Tompa P (2006). Disorder and sequence repeats in hub proteins and their implications for network evolution.. J Proteome Res.

[22] Haynes C, Oldfield CJ, Ji F, Klitgord N, Cusick ME (2006). Intrinsic disorder is a common feature of hub proteins from four eukaryotic interactomes.. PLoS Comput Biol.

[23] Singh GP, Ganapathi M, Dash D (2007). Role of intrinsic disorder in transient interactions of hub proteins.. Proteins.

[24] Iakoucheva LM, Brown CJ, Lawson JD, Obradovic Z, Dunker AK (2002). Intrinsic disorder in cell-signaling and cancer-associated proteins.. J Mol Biol.

[25] Xie H, Vucetic S, Iakoucheva LM, Oldfield CJ, Dunker AK (2007). Functional anthology of intrinsic disorder. 3. Ligands, post-translational modifications, and diseases associated with intrinsically disordered proteins.. J Proteome Res.

[26] Cheng Y, LeGall T, Oldfield CJ, Dunker AK, Uversky VN (2006). Abundance of intrinsic disorder in protein associated with cardiovascular disease.. Biochemistry.

[27] Radivojac P, Obradovic Z, Smith DK, Zhu G, Vucetic S (2004). Protein flexibility and intrinsic disorder.. Protein Science.

[28] Peng K, Radivojac P, Vucetic S, Dunker AK, Obradovic Z (2006). Length-dependent prediction of protein intrinsic disorder.. BMC Bioinformatics.

[29] Romero P, Obradovic Z, Li X, Garner EC, Brown CJ (2001). Sequence complexity of disordered protein.. Proteins.

[30] Radivojac P, Iakoucheva LM, Oldfield CJ, Obradovic Z, Uversky VN (2007). Intrinsic disorder and functional proteomics.. Biophys J.

[31] Lise S, Jones DT (2005). Sequence patterns associated with disordered regions in proteins.. Proteins.

[32] Mohan A, Oldfield CJ, Radivojac P, Vacic V, Cortese MS (2006). Analysis of Molecular Recognition Features (MoRFs).. J Mol Biol.

[33] Fuxreiter M, Tompa P, Simon I (2007). Local structural disorder imparts plasticity on linear motifs.. Bioinformatics.

[34] Neduva V, Russell RB (2006). Peptides mediating interaction networks: new leads at last.. Curr Opin Biotechnol.

[35] Puntervoll P, Linding R, Gemund C, Chabanis-Davidson S, Mattingsdal M (2003). ELM server: A new resource for investigating short functional sites in modular eukaryotic proteins.. Nucleic Acids Res.

[36] Prilusky J, Felder CE, Zeev-Ben-Mordehai T, Rydberg EH, Man O (2005). FoldIndex: a simple tool to predict whether a given protein sequence is intrinsically unfolded.. Bioinformatics.

[37] Linding R, Russell RB, Neduva V, Gibson TJ (2003). GlobPlot: Exploring protein sequences for globularity and disorder.. Nucleic Acids Res.

[38] Garbuzynskiy SO, Lobanov MY, Galzitskaya OV (2004). To be folded or to be unfolded?. Protein Sci.

[39] Dosztanyi Z, Csizmok V, Tompa P, Simon I (2005). The pairwise energy content estimated from amino acid composition discriminates between folded and intrinsically unstructured proteins.. J Mol Biol.

[40] Dyson HJ, Wright PE (2002). Coupling of folding and binding for unstructured proteins.. Current Opinion in Structural Biology.

[41] Sugase K, Dyson HJ, Wright PE (2007). Mechanism of coupled folding and binding of an intrinsically disordered protein.. Nature.

[42] Linding R, Jensen LJ, Diella F, Bork P, Gibson TJ (2003). Protein disorder prediction: implications for structural proteomics.. Structure.

[43] Jones DT, Ward JJ (2003). Prediction of disordered regions in proteins from position specific score matrices.. Proteins: Structure, Function, and Genetics.

[44] Cheng J, Sweredoski MJ, Baldi P (2005). Accurate Prediction of Protein Disordered Regions by Mining Protein Structure Data..

[45] Yang ZR, Thomson R, McNeil P, Esnouf RM (2005). RONN: the bio-basis function neural network technique applied to the detection of natively disordered regions in proteins.. Bioinformatics.

[46] Ward JJ, Sodhi JS, McGuffin LJ, Buxton BF, Jones DT (2004). Prediction and functional analysis of native disorder in proteins from the three kingdoms of life.. Journal of Molecular Biology.

[47] Weathers EA, Paulaitis ME, Woolf TB, Hoh JH (2004). Reduced amino acid alphabet is sufficient to accurately recognize intrinsically disordered protein.. FEBS Lett.

[48] Melamud E, Moult J (2003). Evaluation of disorder predictions in CASP5.. Proteins.

[49] Jin Y, Dunbrack RL (2005). Assessment of disorder predictions in CASP6.. Proteins.

[50] Bordoli L, Kiefer F, Schwede T (2006). Assessment of Disorder Prediction.

[51] Boden M, Bailey TL (2006). Identifying sequence regions undergoing conformational change via predicted continuum secondary structure.. Bioinformatics.

[52] Chen K, Kurgan LA, Ruan J (2007). Prediction of flexible/rigid regions from protein sequences using k-spaced amino acid pairs.. BMC Struct Biol.

[53] Gu J, Gribskov M, Bourne PE (2006). Wiggle-predicting functionally flexible regions from primary sequence.. PLoS Comput Biol.

[54] Liu J, Rost B (2003). NORSp: predictions of long regions without regular secondary structure.. Nucleic Acids Research.

[55] Liu J, Tan H, Rost B (2002). Loopy proteins appear conserved in evolution.. Journal of Molecular Biology.

[56] Moran O, Roessle MW, Mariuzza RA, Dimasi N (2007). Structural features of the full-length adaptor protein GADS in solution determined using small angle X-ray scattering.. Biophys J.

[57] Schlessinger A, Rost B (2005). Protein flexibility and rigidity predicted from sequence.. Proteins.

[58] Schlessinger A, Yachdav G, Rost B (2006). PROFbval: predict flexible and rigid residues in proteins.. Bioinformatics.

[59] Schlessinger A, Punta M, Rost B (2007). Natively unstructured regions in proteins identified from contact predictions.. Bioinformatics.

[60] Esnouf RM, Hamer R, Sussman JL, Silman I, Trudgian D (2006). Honing the in silico toolkit for detecting protein disorder.. Acta Crystallogr D Biol Crystallogr.

[61] Bordoli L, Kiefer F, Schwede T (2007). Assessment of disorder predictions in CASP7.. Proteins.

[62] Obradovic Z, Peng K, Vucetic S, Radivojac P, Dunker AK (2005). Exploiting heterogeneous sequence properties improves prediction of protein disorder.. Proteins.

[63] Mika S, Rost B (2003). UniqueProt: creating representative protein sequence sets.. Nucleic Acids Research.

[64] Sander C, Schneider R (1991). Database of homology-derived protein structures and the structural meaning of sequence alignment.. Proteins.

[65] Rost B (1999). Twilight zone of protein sequence alignments.. Protein Engineering.

[66] Altschul SF, Madden TL, Schaeffer AA, Zhang J, Zhang Z (1997). Gapped BLAST and PSI-BLAST: a new generation of protein database search programs.. Nucleic Acids Research.

[67] Przybylski D, Rost B (2002). Alignments grow, secondary structure prediction improves.. Proteins: Structure, Function, and Genetics.

[68] Romero P, Obradovic Z, Kissinger C, Villafranca JE, Garner E (1998). Thousands of proteins likely to have long disordered regions.. Pac Symp Biocomput.

[69] Rost B, Sander C (1993). Prediction of protein secondary structure at better than 70% accuracy.. Journal of Molecular Biology.

[70] Schlessinger A, Rost B (2005). Protein flexibility and rigidity predicted from sequence.. Proteins: Structure, Function, and Bioinformatics.

[71] Ceroni A, Passerini A, Vullo A, Frasconi P (2006). DISULFIND: a disulfide bonding state and cysteine connectivity prediction server.. Nucleic Acids Res.

[72] Rost B (1994). Conservation and prediction of solvent accessibility in protein families.. Proteins: Structure, Function, and Genetics.

[73] Rost B, Walker JE (2005). How to use protein 1D structure predicted by PROFphd.. The Proteomics Protocols Handbook.

[74] Rost B, Casadio R, Fariselli P, Sander C (1995). Transmembrane helices predicted at 95% accuracy.. Protein Sci.

[75] Rost B, Yachdav G, Liu J (2004). The PredictProtein server.. Nucleic Acids Research.

[76] Rost B (1996). PHD: predicting one-dimensional protein structure by profile based neural networks.. Methods in Enzymology.

[77] Sigalov A, Aivazian D, Stern L (2004). Homooligomerization of the cytoplasmic domain of the T cell receptor zeta chain and of other proteins containing the immunoreceptor tyrosine-based activation motif.. Biochemistry.

[78] Di Lello P, Benison GC, Valafar H, Pitts KE, Summers AO (2004). NMR structural studies reveal a novel protein fold for MerB, the organomercurial lyase involved in the bacterial mercury resistance system.. Biochemistry.

[79] Li M, Song J (2007). The N- and C-termini of the human Nogo molecules are intrinsically unstructured: bioinformatics, CD, NMR characterization, and functional implications.. Proteins.

[80] DiNitto JP, Huber PW (2003). Mutual induced fit binding of Xenopus ribosomal protein L5 to 5S rRNA.. J Mol Biol.

