# Supplementary figures and images for: Improved Disorder Prediction by Combination of Orthogonal Approaches
Source: PLoS One. 2009 Feb 11;4(2):e4433. doi: 10.1371/journal.pone.0004433 (PMC2635965; doi:10.1371/journal.pone.0004433)

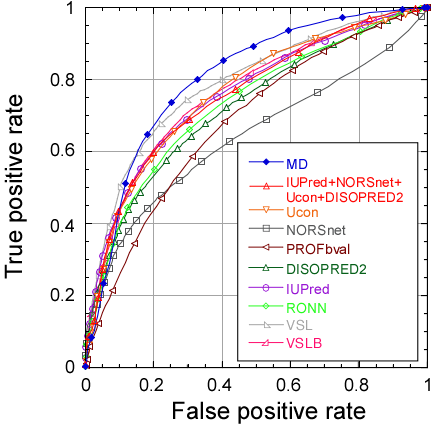

Supplement: Figure S1 — Per-residue performance on sequence-unique DisProt subset using a stringent homology cutoff. ROC curves were compiled using a set with a stricter cutoff for homology redundancy - HSSP-values are <0. The final method MD (blue filled diamonds) that uses neural networks to combine the output of other methods with sequence profiles and other sequence features, is significantly more accurate than the methods that it uses as input such as NORSnet (gray) and DISOPRED2 (dark green) as well as other popular predictors such as IUPred (purple) and RONN (light green). (0.56 MB TIF) [file pone.0004433.s001.tif]

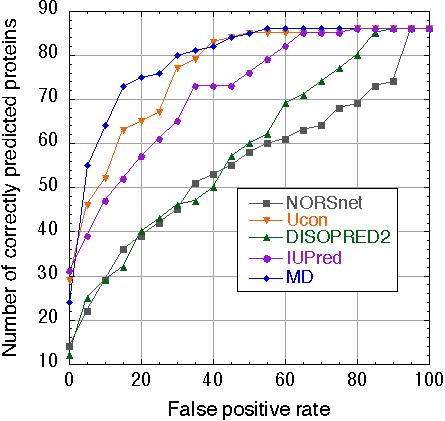

Supplement: Figure S2 — Per-protein performance on long disordered regions. Data set: 86 DisProt proteins with at least one long (>30 residues) disordered region. This set was compiled using more stringent cutoff for homology (HSSP-values<0). Our final method MD identified more true positives than the other methods at most of the false positive rates. Note that this set is much smaller than the one compiled using HSSP-values<10 that the error margins are significantly higher. (0.56 MB TIF) [file pone.0004433.s002.tif]

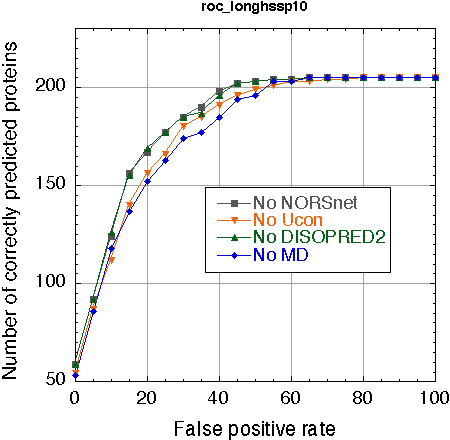

Supplement: Figure S3 — Per-protein performance on long disordered regions when excluding proteins identified by the different methods. Data set: 205 DisProt proteins with at least one long (>30 residues) disordered region. Each line represents the performance when taking protein regions that were correctly identified as disordered by at least one of the methods, while excluding proteins identified by one method. For example, the worst performing combination of three methods is when we did not include MD predictions (blue filled diamonds). (0.59 MB TIF) [file pone.0004433.s003.tif]

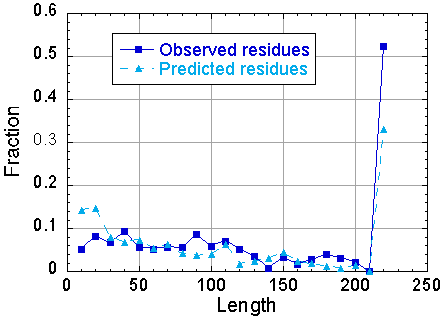

Supplement: Figure S4 — Distribution of observed vs. predicted disordered regions lengths. The fractions of residues that originated from disordered regions from different lengths are plotted. More than 50% of the observed disordered residues originated from very long unstructured regions - regions that are longer than 220 consecutive unstructured residues (dark blue squares), and only about 35% of the predicted residues originated from very long unstructured regions (light blue triangles). Overall, the predictions and observations differed significantly for the two extreme ends of the distribution: MD significantly over-predicted short regions (<30 residues) and significantly under-predicted very long regions. This large difference could be attributed to two main factors; first, among MD's most useful input features was the disorder probability predicted by DISOPRED2. While DISOPRED2 was trained on X-ray disorder, it identifies many short regions as disordered that some were predicted as such by MD as well. This observation gives further evidence that MD captured the flavor of disorder predicted by DISOPRED2. Future improvements of MD may include filtering out very short and isolated predicted stretches. Second, some of the experimental methods characterizing unstructured regions are not accurate enough to determine disorder in a resolution of a few residues. In fact, experimental techniques such as circular dichroism (CD) and analytical ultracentrifugation can only assign disorder at the protein or domain level. (0.42 MB TIF) [file pone.0004433.s004.tif]

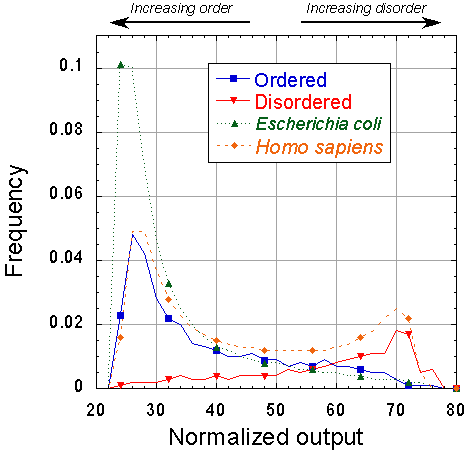

Supplement: Figure S5 — Distribution of method values. The difference between the two neural network output units (one coding for disorder, the other for ordered) was normalized to values ranging from 0 (ordered) to 100 (disordered). Some disordered residues have very low values, i.e. are predicted strongly as well-ordered. These might just be bad, generic prediction mistakes or problems in the original data. Interestingly, residues from E. coli tend to be very low and residues from H. sapiens follow similar distribution to our set. (0.63 MB TIF) [file pone.0004433.s005.tif]
